# Profile of opportunistic infections in AIDS patients in relation to CD4 count in a tertiary care hospital

**DOI:** 10.1186/1471-2334-14-S3-P75

**Published:** 2014-05-27

**Authors:** AR Gnananjali, MN Sumana

**Affiliations:** 1 Department of Microbiology, JSS Medical College, Mysore, Karnataka, India

## Background

HIV/AIDS is a global pandemic with cases reported from virtually every country. The CD4 cells are the primary target cells for HIV. The hallmark of HIV disease is a profound immunodeficiency resulting from a progressive quantitative and qualitative deficiency of CD4+ T cells. As CD4 count decreases, opportunistic infections (OIs) manifest. HIV infection progressing to AIDS is associated with many OIs.

## Methods

One hundred seropositive HIV patients were selected, depending upon clinical presentation and physical examination, appropriate laboratory diagnostic methods were employed for detection of OIs. CD4 count was done by FACS. The occurrence of various OIs and CD4 counts were correlated.

## Results

This study involved a descriptive study and tuberculosis (43.9 %) was found to be most common OI seen in patients with mean CD4 count 360 ± 56cells /µL , followed by candidiasis (14.6 %) with mean CD4 count 324 ± 42cells/µL , diarrhea due to coccidian parasite infection (8.5%) with mean CD4 count 97±15 cells/µL, cryptococcal meningitis (3.65 %) and *Pneumocystis jiroveci* pneumonia (3.65 %) with mean CD4 count 92 ± 6 cells/µL. CD4 count of 20 patients were followed up 6 months after initiating ART and it was observed that CD4 count in 16 patients had increased.

## Conclusion

Tuberculosis and candidiasis was seen in patients with CD4 count above 300cells/µL. Diarrhea due to coccidian parasites, cryptococcal meningitis and PCP was seen in patients with CD4 count below 100cells/µL. OIs cause substantial morbidity and hospitalization, and shortens survival of HIV infected patients.

